# Motivation to treatment-seeking among individuals with addiction to benzodiazepines: a qualitative study

**DOI:** 10.1192/j.eurpsy.2025.835

**Published:** 2025-08-26

**Authors:** C. Krüger, S. Burmester, J. Franck, M. Hedlund Lindberg, J. Westman

**Affiliations:** 1 Department of Neurobiology, Care Sciences and Society, Karolinska Institutet, Huddinge; 2 The Stockholm Centre for Dependency Disorders, Stockholm Health Care Services, Region Stockholm; 3 Department of Clinical Neuroscience, Karolinska Institutet, Stockholm; 4 Department of Medical Sciences, Psychiatry, Uppsala University, Uppsala; 5 Academic Primary Care Centre, Region Stockholm; 6 Department of Health Care Sciences, Marie Cederschiöld University, Stockholm, Sweden

## Abstract

**Introduction:**

Many individuals with long-term use benzodiazepines and benzodiazepine-like hypnotics, commonly prescribed for the short-term management of anxiety or sleeping problems, develop addiction. It is therefore important to better understand what motivates individuals with addiction to quit. Few prior qualitative studies have explored patients’ perceptions and experiences of addiction to benzodiazepines in the context of motivation to seek treatment.

**Objectives:**

This study explored how individuals perceive addiction to benzodiazepines and aimed to describe the experiences that motivated them to seek treatment.

**Methods:**

This exploratory qualitative study was conducted among nineteen adults (≥18 years) diagnosed with sedative, hypnotic, or anxiolytic use disorder. Participants were purposively recruited from a publicly funded outpatient addiction clinic in Sweden and were undergoing tapering treatment at the time of their in-depth interviews. The interviews, which followed a semi-structured guide, were completed between April 2021 and February 2023. Transcripts were analyzed using reflexive thematic analysis by a multidisciplinary team. All participants provided written informed consent and the study was approved by the Swedish Ethical Review Authority (Dnr. 2019-05302).

**Results:**

Participants described perceptions of addiction and motivation to quit in terms of the growing harms they experienced from continued use of benzodiazepines. We identified three themes that reflect the nonlinear process and multifaceted consequences which to a “tipping point” where individuals made the decision to seek treatment. Theme one described how benzodiazepine use required increasing mental energy and time, and how participants felt their freedom was restricted by the need to “hunt” for medication. In theme two, participants described facing a crossroads regarding their benzodiazepine use as the effectiveness decreased. Some participants defined addiction in relation to the negative cycle of dose escalation and withdrawal symptoms they experienced, which also motivated them to seek treatment. Theme three discusses the ways that benzodiazepines negatively impacted different psychosocial and practical aspects of daily life, including conflicts related to changes in personality and negative impacts on relationships, which resulted in further areas of motivation.

**Conclusions:**

The results suggest that individuals with addiction to benzodiazepines reach the decision to seek treatment both through processes of change over time and through individual factors that act as a tipping point. These motivational factors are of clinical importance in the decision to seek treatment and should be identified by healthcare providers and cultivated in individuals with addiction to benzodiazepines.

**Disclosure of Interest:**

None Declared

